# Mobility and Generation of Mosaic Non-Autonomous Transposons by Tn*3*-Derived Inverted-Repeat Miniature Elements (TIMEs)

**DOI:** 10.1371/journal.pone.0105010

**Published:** 2014-08-14

**Authors:** Magdalena Szuplewska, Marta Ludwiczak, Katarzyna Lyzwa, Jakub Czarnecki, Dariusz Bartosik

**Affiliations:** Department of Bacterial Genetics, Institute of Microbiology, Faculty of Biology, University of Warsaw, Warsaw, Poland; University of Manchester, United Kingdom

## Abstract

Functional transposable elements (TEs) of several *Pseudomonas* spp. strains isolated from black shale ore of Lubin mine and from post-flotation tailings of Zelazny Most in Poland, were identified using a positive selection trap plasmid strategy. This approach led to the capture and characterization of (i) 13 insertion sequences from 5 IS families (IS*3*, IS*5*, IS*L3*, IS*30* and IS*1380*), (ii) isoforms of two Tn*3*-family transposons – Tn*5563*a and Tn*4662*a (the latter contains a toxin-antitoxin system), as well as (iii) non-autonomous TEs of diverse structure, ranging in size from 262 to 3892 bp. The non-autonomous elements transposed into AT-rich DNA regions and generated 5- or 6-bp sequence duplications at the target site of transposition. Although these TEs lack a transposase gene, they contain homologous 38-bp-long terminal inverted repeat sequences (IRs), highly conserved in Tn*5563*a and many other Tn*3*-family transposons. The simplest elements of this type, designated TIMEs (Tn*3* family-derived Inverted-repeat Miniature Elements) (262 bp), were identified within two natural plasmids (pZM1P1 and pLM8P2) of *Pseudomonas* spp. It was demonstrated that TIMEs are able to mobilize segments of plasmid DNA for transposition, which results in the generation of more complex non-autonomous elements, resembling IS-driven composite transposons in structure. Such transposon-like elements may contain different functional genetic modules in their core regions, including plasmid replication systems. Another non-autonomous element “captured” with a trap plasmid was a TIME derivative containing a predicted resolvase gene and a *res* site typical for many Tn*3*-family transposons. The identification of a portable site-specific recombination system is another intriguing example confirming the important role of non-autonomous TEs of the TIME family in shuffling genetic information in bacterial genomes. Transposition of such mosaic elements may have a significant impact on diversity and evolution, not only of transposons and plasmids, but also of other types of mobile genetic elements.

## Introduction

Transposable elements (TEs) play a very important role in bacterial evolution. They can shape the structure of bacterial genomes and influence the expression of many genes by the generation of various DNA rearrangements. The presence and activity of TEs may lead not only to insertional inactivation of random genes, but also to deletions, duplications and translocations of DNA segments as well as replicon fusions. Moreover, some elements contain promoters that can drive transcription of downstream genes, which may result in the activation and expression of various previously silent phenotypes [1].

TEs are highly diverse with respect to their structure, specific properties and the regulation of the transposition process. Due to their genetic organization they may be divided into two transposon classes. Class I groups insertion sequences (ISs) – the simplest TEs – and elements whose transposition is dependent on ISs, i.e. (i) composite transposons containing two flanking IS copies, and (ii) transposable modules (TMos) [2], [3] or ISCR (Insertion Sequence Common Region) [4], containing only one terminally-placed IS copy. Class II transposons, gathering mainly elements of the Tn*3* and Tn*7* families, encode a more complex transposition machinery.

The characteristic feature of all these elements is the presence of a gene encoding transposase: an enzyme that catalyzes the process of transposition. Analyses performed by Aziz and colleagues revealed that transposase genes are the most prevalent genes in nature, which illustrates the huge scale and rate of transposition events leading to the acceleration of biological diversification [5]. Transposases specifically recognize and interact with the terminal parts of TEs, which in most cases carry inverted repeat sequences (IRs). Transposase accessibility is therefore the main factor that determines the frequency of transposition. Many of the identified regulatory mechanisms that control the process of transposition act at the level of transcription or translation of the proteins involved [6]. Many transposases preferentially act *in cis*, since they more frequently bind DNA at their site of synthesis. Therefore, the frequency of transposition is inversely proportional to the distance between the transposase gene and its cognate binding site. Nevertheless, activation of transposition *in trans* is not a rare phenomenon (as shown e.g. in the case of several ISs as well as Tn*5*- and Tn*3*-family elements) and may favor the movement of not only intact elements, but also of mobile insertion cassettes (MICs) [7] and non-autonomous miniature inverted-repeat TEs (MITEs).

MITEs are defined as small (approx. 30–300 bp) multicopy elements that are present in up to several hundred copies per genome. They contain terminal inverted repeats, often similar in sequence to IRs of intact ISs [8]. However, their core regions lack a transposase gene. In many cases these elements are bordered by short direct repeats (DRs) (usually the dinucleotide TA), so in this respect they resemble functional TEs whose transposition leads to duplication of the insertion target sequence [9]. The aforementioned features strongly suggest that MITEs originate from defective ISs and their transposition may be trans-activated by compatible transposases [10]. However, mobility of these elements has been demonstrated in only a very few cases (e.g. [11]).

The first prokaryotic MITEs were discovered in *Neisseria* spp. (CREE – Correia Repeat Enclosed Element) [12], [13]. Bioinformatic analyses have since led to the identification of diverse types of MITE occurring in different taxonomic groups of bacteria [8]. Such elements are found in intergenic and intragenic genomic locations. They are often co-transcribed with the upstream genes, which may influence the conformation and stability of the resulting transcripts [14]. Moreover, some MITEs contain, in their core regions, functional promoters and diverse sequence motifs recognized by host proteins (e.g. integration-host-factor binding site), which may additionally modulate the expression of nearby genes [8]. These elements may therefore play important regulatory roles. Furthermore, the presence of many identical MITEs in a cell can lead to genetic rearrangements resulting from homologous recombination, which may influence the genome architecture.

In this study, we identified and characterized functional autonomous as well as non-autonomous transposable elements originating from transposons of the Tn*3* family. We have demonstrated their transposition activity and ability to mobilize genomic DNA for transposition, which leads to the generation of diverse non-autonomous elements of more complex structure. The obtained results shed new light on the diversity and properties of TEs of the Tn*3* family, which are the most ubiquitous transposons in bacteria.

## Materials and Methods

### Bacterial strains, plasmids and culture conditions

The bacterial strains analyzed in this study were isolated from black shale ore collected in Lubin copper mine (LM) (51°24′N–16°12′E) and postflotation tailings sampled from Zelazny Most (ZM) (51°30′N–16°12′E) in Poland, during the Bioshale FP6 European project [15]. The samples used for strain isolation were provided by the Copper Research and Design Centre Ltd. Research and Development Centre, being a part of KGHM POLSKA MIEDŹ SA capital group (owner of the Lubin mine). The field studies did not involve endangered or protected species. The strains (listed in Table S1) were classified by 16S rDNA sequence similarity. Rifampicin resistant (Rif^r^) derivatives of the wild-type strains of *Pseudomonas* spp. were obtained and used as host strains for trap plasmid pMAT1. *Escherichia coli* TG1 [16] was used in plasmid construction. Bacteria were grown in Luria-Bertani (LB) medium [16] at 37°C (*E. coli*) or 30°C (other strains). When necessary, the medium was supplemented with sucrose (10%) and with antibiotics: ampicillin (100 µg/ml), kanamycin (300 µg/ml - *Pseudomonas aeruginosa* LM10; 50 µg/ml - other strains), rifampicin (50 µg/ml). The plasmids used in this study have been described previously: (i) pMAT1 (Km^r^, *ori* pBBR1, *oriT* RK2, *sacB*) [3] and (ii) pCM132 (Km^r^, *ori* pMB1, *ori* RK2, *oriT* RK2, *lacZ*) [17], employed for the identification of TEs and β-galactosidase assays, respectively.

### DNA manipulations

Common DNA manipulation methods were performed as described by Sambrook and Russell [16]. Plasmid DNA was isolated using a standard alkaline lysis procedure [18] and, when required, purified by CsCl-ethidium bromide density gradient centrifugation. Total genomic DNA of *Pseudomonas* spp. was isolated by a standard procedure [19].

### Identification of a pool of transposable elements (TEs)

The *E. coli*-*Pseudomonas* spp. shuttle vector pMAT1 was used for the identification of TEs [3]. This trap plasmid contains the *sacB* gene of *Bacillus subtilis*, coding for levan sucrase, an enzyme that catalyzes sucrose hydrolysis and levan extension. The expression of *sacB* is lethal for Gram-negative bacteria in the presence of sucrose [20]. Therefore, cells carrying the functional *sacB* gene are sucrose sensitive (Suc^s^) and their cultivation in medium containing sucrose results in cell lysis. This allows direct selection of *sacB* mutants (Suc^r^) (e.g. carrying inserted TEs), whose growth is not affected under these conditions, thus enabling positive selection of transposition mutants.

### Introduction of plasmid DNA into bacterial cells

DNA was introduced into *Pseudomonas* spp. by triparental mating as described previously [21], or into *E. coli* by chemical transformation according to the method of Kushner [22].

### PCR amplification

Relevant DNA regions were amplified by PCR using appropriate template DNAs, specific oligonucleotide primers (listed in Table S2), dNTPs and DreamTaq Green PCR Master Mix (Thermo Scientific) or Taq DNA polymerase (Qiagen, with supplied buffer) in a Mastercycler (Eppendorf). Products of PCR amplification were separated by electrophoresis on 0.8 or 2% agarose gels and purified with a QIAquick PCR Purification Kit (Qiagen).

### DNA-DNA hybridization

The copy number and genomic localization of the identified TEs as well as the distribution of homologous sequences in the *Pseudomonas* spp. genomes were analyzed by DNA-DNA hybridization. Molecular probes specific for TIME elements were prepared by (i) PCR amplification of selected DNA fragments (specific oligonucleotide primer pairs are listed in Table S2) and (ii) labelling of gel-purified amplified DNA fragments with digoxigenin (DIG, Roche). Total genomic DNA or plasmid DNA of the analyzed strains were digested using appropriate restriction enzymes (to avoid multiple hybridization signals from a single copy of a given TE) and, after DNA electrophoresis, transferred onto nylon membrane (Roche). Hybridization and visualization of bound DIG-labelled probes were carried out as recommended by the supplier. The number of DNA bands hybridizing with each probe was equivalent to the minimum number of copies of a given element within the genome.

### Assay for β-galactosidase activity

DNA fragments containing a TIME1 element or a 3′-end fragment of TIME-RES were amplified by PCR with appropriate primer pairs (listed in Table S2) and cloned into the promoter-probe vector pCM132 [17] upstream of the promoter-less *lacZ* reporter gene. Promoter activity was confirmed by assays for β-galactosidase activity in *P. aeruginosa* PAO1161 as described by Thibodeau and colleagues [23]. The growth of bacterial cultures and kinetic β-galactosidase assays were performed using 96-well plates. Individual assays were repeated three times.

### Sequence analyses and annotation

The nucleotide sequences of identified transposable elements were determined using a dye terminator sequencing kit and an automatic sequencer (ABI 377 Perkin Elmer). Similarity searches were performed using the ISfinder (http://www-is.biotoul.fr/is.html) [24] and BLAST programs [25] provided by the National Center for Biotechnology Information (http://www.ncbi. *nlm.nih.gov/BLAST/*).

### Nucleotide sequence accession numbers

Nucleotide sequences obtained in this study have been deposited in the GenBank database. Nucleotide sequences of the identified insertion sequences have been additionally deposited in the ISfinder database. GenBank accession numbers of the 16S rDNA sequences of *Pseudomonas* spp. strains are given in Table S1. Accession numbers of the nucleotide sequences of TEs are given in Table 1. The accession numbers of the plasmid pZM1P1 and pLM8P2 nucleotide sequences are KJ940992 and KJ940994, respectively.

**Table 1:** Characteristics of transposable elements identified in this study.

Class of TE	TE	TE (family/group)	Length (bp)	IR (bp)^a^	DR (bp)	GC (%)	Frequency of transposition (host strain)	Accession number
**IS**	**IS** ***Pen2*** **a**	**IS** ***3*** **/IS** ***51***	1232	26/25	3	55.0	7.3×10^−7^ (LM15)	KJ940995
	**IS** ***Pa8*** **a**	**IS** ***5*** **/IS** ***5***	1324	18/18	4	63.9	4.5×10^−7^ (LM14)	KJ920389
	**IS** ***Pre1*** **a**	**IS** ***5*** **/IS** ***5***	1192	16/12	4	57.3	4.8×10^−6^ (LM12)	KJ920390
	**IS** ***Pre2*** **a**	**IS** ***5*** **/IS** ***5***	1190	16/11	4	57.3	4.4×10^−4^ (LM8)	KJ920391
	**IS** ***Psp6***	**IS** ***5*** **/IS** ***5***	1210	22/18	4	57.8	7.7×10^−7^ (ZM1)	KJ920383
	**IS** ***Psp10***	**IS** ***5*** **/IS** ***427***	942	21/14	2	57.2	6.7×10^−7^ (LM25)	KJ920388
	**IS** ***1394*** **a**	**IS** ***30***	1100	27/23	3	62.5	3.6×10^−5^ (LM14)	KJ920392
	**IS** ***Psp7***	**IS** ***30***	1113	27/21	5	60.6	1.9×10^−4^ (ZM2)	KJ920384
	**IS** ***Psme1***	**IS** ***30***	1064	25/21	5	57.0	4.3×10^−6^ (LM7)	KJ920385
	**IS** ***Psp8***	**IS** ***30***	1113	27/22	3	60.6	1.8×10^−5^ (LM13)	KJ920386
	**IS** ***Pa33*** **a**	**IS** ***1380***	1561	16/16	4	60.8	4.3×10^−7^ (LM7)	KJ920393
							1.6×10^−6^ (LM13)	
	**IS** ***Psp9***	**IS** ***1380***	1718	21/18	5	63.6	1.6×10^−6^ (LM13)	KJ920387
	**IS** ***Ppu12*** **a**	**IS** ***L3***	3372	24/21	8	56.0	4.5×10^−7^ (LM14)	KJ920394
**Tn**	**Tn** ***4662*** **a**	**Tn** ***3***	7196	41/36	5	60.6	2.5×10^−5^ (LM10)	KJ920396
							9.8×10^−6^ (LM13)	
	**Tn** ***5563*** **a**	**Tn** ***3***	6253	39/39	5	62.2	2.7×10^−5^ (LM7)	KJ920395
**MITE**	**TIME1**	**Tn** ***3***	262	38/29	6	54.2	2.3×10^−6^ (ZM1)	KJ920397
	**TIME2**	**Tn** ***3***	262	38/30	5/6	53.4	1.4×10^−4^ (LM8)	KJ920398
	**TIME3**	**Tn** ***3***	262	38/29	5	53.1	7.1×10^−6^ (LM8)	KJ920399
	**TIME4**	**Tn** ***3***	262	38/29	nd	53.8	nd	KJ940992
	**TIME-RES**	**Tn** ***3***	1186	40/32	5	61.6	7.8×10^−5^ (LM8)	KJ934990
	**TIME-COMP1**	**Tn** ***3***	2469	38/38	5	53.3	6.0×10^−5^ (ZM1)	KJ940992
	**TIME-COMP2**	**Tn** ***3***	3892	38/29	5	65.5	7.7×10^−7^ (ZM1)	KJ940993

a The length of the IRs/the number of identical residues. nd - not determined.

## Results

### Isolation and identification of Pseudomonas spp. strains

The bacterial strains analyzed in this study were isolated from black shale ore from the active Lubin copper mine and from postflotation tailings from Zelazny Most reservoir (Lower Silesia province, Poland) [26]. The copper deposit in this region is polymetallic and contains a particularly high concentration of valuable metals [27]. The chemical composition of the black shale ore makes it a unique and extreme environment for living organisms because of (i) the low amount of easily degradable carbon sources, (ii) high concentration of heavy metals and arsenic, (iii) alkaline pH and (iv) high salinity [28], [29].

Twenty-six bacterial strains were isolated, half of which were classified as members of the genus *Pseudomonas* (*Gammaproteobacteria*) by 16S rDNA sequence similarity. Two of the strains were identified at the species level (Table S1). The *Pseudomonas* spp. strains, designated LM5-LM8, LM10-LM15, LM25 (LM – Lubin Mine) and ZM1, ZM2 (ZM – Zelazny Most), were subjected to more detailed analysis aimed at characterizing their TEs.

### Identification of a pool of TEs

To identify functional TEs within 13 *Pseudomonas* spp. strains we employed the trap plasmid pMAT1, containing the *sacB* gene of *B. subtilis* as a positive-selection cassette [3]. After introducing the plasmid by triparental mating into rifampicin resistant (Rif^r^) derivatives of the aforementioned strains (denoted LM5R, LM6R, etc.), transposition events, which occurred within the *sacB* cassette, were selected by the application of LB agar medium supplemented with sucrose (selection of Suc^r^ clones; see Materials and Methods for details). The plasmids carried by one hundred Suc^r^ colonies of each strain were analyzed by DNA electrophoresis. This revealed that approx. 45% of the examined clones carried pMAT1-derivatives containing inserts of different size: (i) <1 kb, (ii) 1–2 kb and (iii) >2 kb. The location of the TEs within the *sacB* gene was confirmed by PCR using a set of previously described cartridge-specific primers [3]. Further analysis (DNA restriction mapping, DNA sequencing and DNA hybridization) confirmed the “capture” of functional transposable elements, whose characteristics are presented in Table 1.

### Genetic structure of the TEs

**Figure 1:**
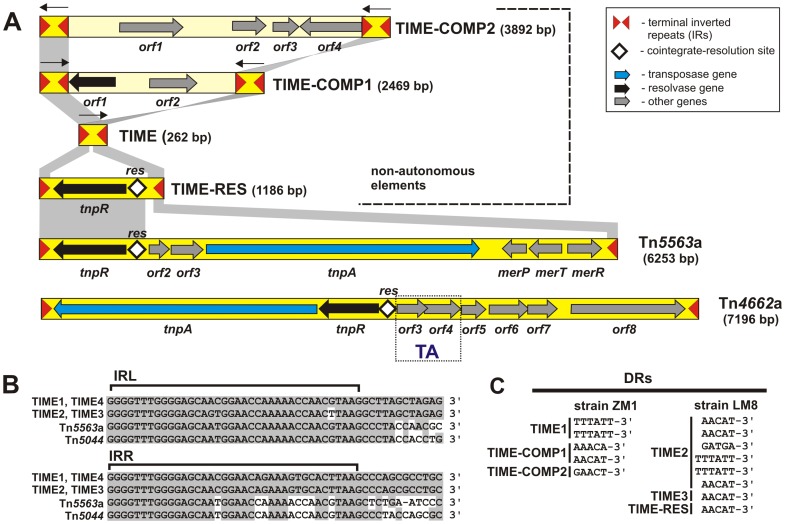
Non-autonomous and autonomous transposable elements of the Tn*3* transposon family identified in *Pseudomonas* spp. strains. A. The genetic organization of TEs identified with trap plasmid pMAT1 in strains ZM1 (TIME, TIME-COMP1, TIME-COMP2), LM8 (TIME, TIME-RES), LM10 and LM13 (Tn*4662*a), as well as LM7 (Tn*5563*a). Inverted repeats (IRs) flanking the elements are marked by red triangles. Predicted coding regions are represented by thick arrows indicating the direction of transcription. Thin black arrows above TIMEs indicate the orientation of the elements. Shaded areas connect homologous DNA regions. *res* indicates the cointegrate resolution site of the site-specific recombination systems. The predicted toxin-antitoxin system is boxed and denoted by TA. Note that TIMEs are not drawn to scale. B. Alignment of the terminal inverted repeat nucleotide sequences (IRL – left IR; IRR – right IR) of TIMEs and two autonomous Tn*3*-family transposons, Tn*5563*a and Tn*5044*. Identical residues are indicated by gray shading. C. Nucleotide sequences of direct repeats (DRs) generated by TIME elements during transposition into the selective cartridge of pMAT1.

Detailed analysis of the nucleotide sequences of the “captured” elements characterized their genetic structure by identifying (in most clones) (i) the transposase gene, (ii) terminal inverted repeats (IR), being the sites for transposase binding and action, and (iii) the target sequence (DR), which in most cases was duplicated upon insertion. The elements were classified into appropriate IS/Tn families based on the results of comparative analysis. This confirmed the identification of 13 insertion sequences (ISs) and several elements of the Tn*3* transposon family, including a novel type of non-autonomous element of mosaic structure (Fig. 1).

### Characterization of the ISs

As shown in Table 1, the “captured” ISs include representatives of five IS families. The most numerous are members of the IS*5* and IS*30* families. Among 13 identified ISs, six are novel elements, which have been given the following designation: (i–ii) IS*Psp6* (host strain ZM1) and IS*Psp10* (LM25) – IS*5* family (IS*5* and IS*427* groups, respectively), (iii–v) IS*Psp7* (ZM2), IS*Psme1* (LM7) and IS*Psp8* (LM13) – IS*30* family, and (vi) IS*Psp9* (LM13) – IS*1380* family. All of the other identified ISs are isoforms of previously described elements (Table 1) (according to the ISfinder database isoforms show more than 98% amino acid sequence similarity between transposases and/or more than 95% of identity between entire IS nucleotide sequences).

All but one IS shows the greatest similarity to elements previously identified in various *Pseudomonas* spp. strains (data not shown). The one exception is IS*Psp9* (IS*1380* family) whose transposase is most similar [approx. 27% amino acid (aa) sequence identity] to transposases of two Gram-positive bacteria: *Streptococcus suis* 05ZYH33 (IS*Ssu5*) and *Bacillus halodurans* C-125 (IS*Bha1*) (accession nos. NC_009442 and AB024553, respectively).

Most of the “captured” elements contain only one open reading frame (ORF), encoding a transposase. IS*Pen2*a and IS*Psp10* (Table 1) have two ORFs encoding predicted trans-frame fusion transposases, which is common among members of the IS*3* (group IS*51*) and IS*5* (group IS*427*) families [9]. A unique feature of IS*Psp10* is the lack of a stop codon within ORF2 encoding the terminal part of the transposase. IS*Psp10* transposed into the *sacB* gene just upstream of a +TAA sequence (upon insertion the dinucleotide TA was duplicated); therefore, the stop codon in this case is provided by the target site of transposition. It is likely that IS*Psp10* preferentially recognizes such target sites in order to remain active, although due to the very low transposition frequency of this element, we were unable to analyze other transposition events.

Of particular interest is IS*Ppu12*a (IS*L3* family), which resembles non-composite transposons in structure. Besides *tnpA* (transposase) and *tnpR* (transcriptional regulator) genes, this element contains two additional ORFs not involved in the process of transposition, encoding a putative lipoprotein signal peptidase and divalent heavy metal antiporter [30].

### Characterization of the Tn3-family elements

#### Autonomous elements

In the tested strains we identified two functional non-composite transposons of the Tn*3* family. These are isoforms of previously described elements: (i) Tn*4662*– distinguished *in silico* from the nucleotide sequence of *Pseudomonas putida* HS1 plasmid pDK1 [31] and (ii) Tn*5563* identified from the sequence of plasmid pRA2 of *Pseudomonas alcaligenes* NCIB 9867 [32] (Table 1).

Transposons Tn*4662*a (captured in LM10 and LM13 strains) and Tn*5563*a (LM7) are not highly related, since they represent two subfamilies of the Tn*3* family (Tn*501* and Tn*5044*, respectively). These elements differ in overall genetic structure and their transposases show only 21% aa sequence similarity. As is typical for Tn*3*-family transposons, both elements contain a site-specific recombination module (RES) encoding a resolvase (TnpR) responsible for the resolution of cointegrates (at the *res* sites), which are intermediate forms in replicative transposition.

Tn*5563*a has identical 39-bp-long IRs, whose sequence shows significant similarity to IRs of Tn*3*, Tn*501* and Tn*5044*, the archetypes of three distinguished subfamilies of the Tn*3* transposon family. In contrast, the IRL and IRR of Tn*4622*a (41 bp) are not identical (nucleotide differences at 5 positions) and their similarity to IRs of other Tn*3* family members is limited to short sequence stretches (data not shown). Both elements transposed into A+T-rich sequence regions of the *sacB* gene, and generated 5-bp DRs: 5′-AAATT-3′ (LM10), 5′-AAATA-3′ (LM13) and 5′-AATAT-3′ (LM7).

As shown in Fig. 1A, Tn*4662*a contains eight ORFs. Besides the transposase and resolvase genes (ORF1 and ORF2) it also encodes three putative proteins of unknown function (ORF6-ORF8) as well as components of a toxin-antitoxin system (ORF3 and ORF4). The putative *orf3-orf4* operon is highly homologous to the *tad*-*ata* addiction system identified in plasmid pAMI2 of *Paracoccus aminophilus* JCM 7686 [33]. Orf3 protein of Tn*4662*a shares 73% aa identity with the Tad toxin (YP_001965064), while Orf4 is 70% identical to antitoxin Ata, a member of the XRE/CRO family (YP_001965065). Homologous loci are also present within transposon Tn*4662* as well as closely related Tn*5501*-Tn*5502* [34] and Tn*5503* [35], but they were not distinguished at the time of sequence deposition. Such portable genetic modules (when functional) may potentially provide stabilization to the transposons and their carrier replicons by eliminating TA-less clones from the bacterial population.

The second transposon identified in this study, Tn*5563*a (Fig. 1A), is a member of a Tn*3* subfamily whose archetype is Tn*5044* of *Xanthomonas campestris* (accession no. Y17691) [35]. These transposons contain highly homologous recombination systems that mediate the process of transposition and resolution of co-integrates, as well as mercury resistance operons (*mer*). Tn*5044* carries a complete set of 7 *mer* genes (also conserved in other transposons, e.g. Tn*21* and Tn*501*), while Tn*5563*a contains only part of this operon: genes *merP* and *merT* encoding mercury transporters, and *merR* encoding a transcription regulator (ORF5-ORF7; Fig. 1A).

#### Non-autonomous elements

Detailed analysis revealed that some of the pMAT1 derivatives isolated from Suc^r^ clones of the ZM1 and LM8 strains contained short DNA inserts not exceeding 300 bp in size within the *sacB* gene. The nucleotide sequence of one of these inserts was obtained. An internal part of the insert was then amplified by PCR, labeled with digoxigenin (DIG) and used as a molecular probe in hybridization analysis of all pMAT1 derivatives obtained in the ZM1 and LM8 strains. This analysis revealed that small homologous DNA inserts were present in 3 ZM1 plasmids and in 18 LM8 plasmids. Interestingly, plasmids from both strains containing larger insertions were also detected. These inserts fall within 3 size ranges: (i) 1–2 kb (1 plasmid in LM8), (ii) 2–3 kb (78 plasmids in ZM1) and (iii) 3–4 kb (1 plasmid in ZM1). PCR analysis using four cassette-specific primers [3] confirmed that all the insertions occurred within the *sacB* gene of pMAT1. Further analysis showed that the identified DNA segments represent two types of non-autonomous TEs of the Tn*3* family (Fig. 1A).

**Figure 2:**
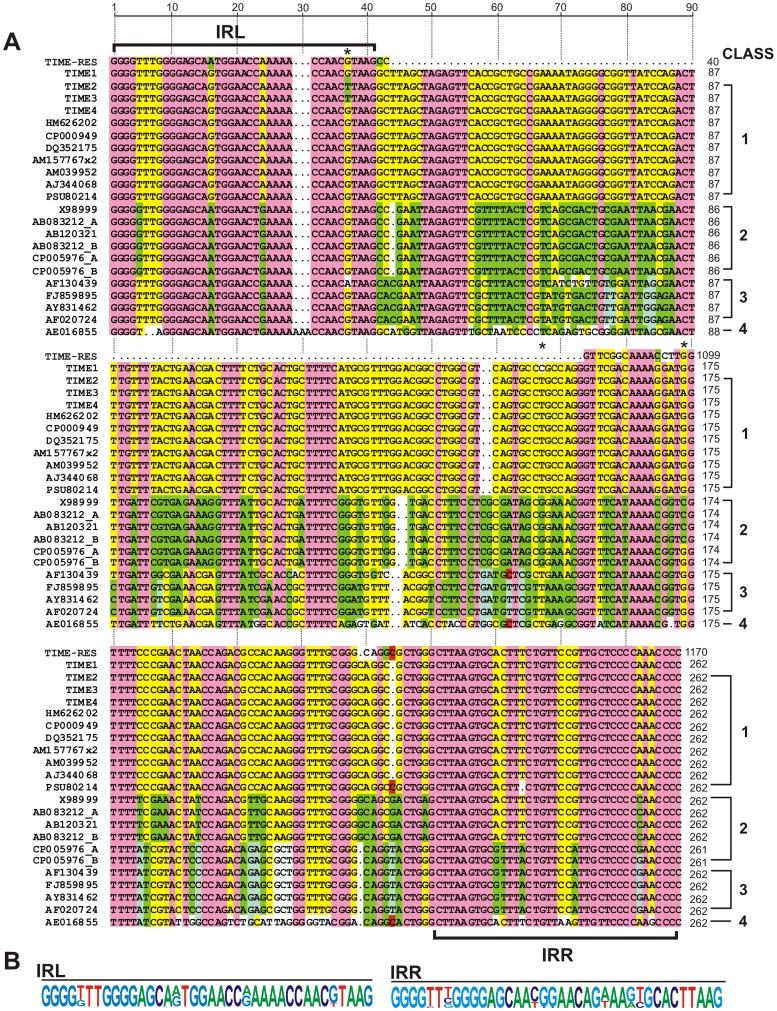
Comparison of the nucleotide sequences of TIME elements. A. Multiple alignment of the nucleotide sequences of TIMEs identified in bacterial genomes (NCBI database) by *in silico* comparative analyses. Accession numbers of the sequences are given on the left. A detailed description of the sequences (including the host strain, replicon and nucleotide positions) is presented in Table S3. TIME1-TIME4– functional non-autonomous elements identified in this study (stars indicate nucleotide substitutions in the TIME2-TIME4 sequences in relation to TIME1). Dots indicate gaps introduced to optimize the alignment. Only the sequence of TIME-RES homologous to TIME was used for the comparative analysis. Residues conserved in all predicted elements are shaded light violet, residues conserved in TIME1-TIME4 and other elements are shaded yellow, and those conserved in other elements (but not in TIME1-TIME4) are shaded green and red. The IRL and IRR sequences are marked. Four classes of TIME, distinguished by sequence similarity, are indicated on the right. B. Comparative analysis of the TIME and TIME-RES IR sequences shown as a pictogram (font size corresponds to the relative frequencies of the bases at each position within the termini). Numbers at right indicate the position in nucleotide sequence of each element.

The nucleotide sequences of 9 the smallest “captured” elements were determined (2 from ZM1 and 7 from LM8). All of these elements were of the same size (262 bp) and they showed a very high level of sequence identity. The elements identified in ZM1 were identical, and those of LM8 differed only in 2 nucleotides: a transversion of guanine to thymine at position 34 (IRL) and a transition of cytosine to thymine at position 152 (Fig. 2A, asterisks mark variable positions). Moreover, among the elements “captured” in strain LM8, a single isoform was found with an additional nucleotide change (a guanine to adenine transition at position 174) (Fig. 2A).

The core region of these elements shows no sequence similarities to any other transposable element described so far. They contain no ORFs, and therefore they do not encode a transposase. At both ends they contain 38-bp terminal IRs sharing a significant level of sequence identity with IRs of transposons of the Tn*5044* subgroup of the Tn*3* transposon family. The highest identity was found with terminal repeats of transposons Tn*5563*a (identified in this study) and Tn*5044* (Fig. 1B).

All of these elements are bordered by short (5- or 6-bp long) duplicated AT-rich target sequences (DRs) (Fig. 1C), confirming their transposition into the selective cartridge of pMAT1. Interestingly, all the 6-bp DRs in the elements from both strains are identical (5′-TTTATT-3′) and resulted from transposition into the same site within the *sacB* gene. This suggests that the generation of DRs of this size may be dependent on a specific target sequence (Fig. 1C).

Since the identified elements do not encode a transposase, their transposition has to be dependent on *trans*-encoded transposase. The presence of IRs typical for some members of the Tn*3* family strongly suggests that these elements are mobilized for transposition by *trans*-encoded Tn*3*-family transposases. They are therefore non-autonomous elements, which may be included in the group of miniature inverted-repeat transposable elements (MITEs). Based on the IR sequence homology, we propose that the elements identified in this study are designated as TIMEs (**T**n*3*-derived **I**nverted-repeat **M**iniature **E**lements). The three identified isoforms have been named TIME1 (ZM1 strain), TIME2 and TIME3 (LM8 strain) (Fig. 2A).

The larger “captured” elements were transposon-like non-autonomous TEs. These elements appear to have originated from one (TIME-RES, i.e. resolutive TIME) or two TIMEs (TIME-COMP, i.e. composite TIME) (Table 1) (Fig. 1A).

TIME-RES (ZM1 strain; 1186 bp; G+C content 61.6%) transposed into the *sacB* gene of pMAT1 at a frequency of 7.8×10^−5^. Its insertion resulted in the duplication of the 5-bp target sequence 5′-AACAT-3′. TIME-RES has imperfect terminal IRs of 40 bp (with 8 mismatches), with high sequence similarity (95%) to the IRs of TIMEs (Fig. 1B). The sequence conservation of TIME-RES and TIMEs is also observed within a 60 bp-long DNA region adjacent to the IRR (Fig. 2) (94% sequence identity).

The major part of the core region of TIME-RES is composed of an ORF encoding a putative resolvase TnpR (309 aa) as well as an AT-rich putative recombination site (*res*). This DNA region displays 99% nucleotide sequence identity to the co-integrate resolution system of the mercury-resistance transposon Tn*5563* [32] (as well as to the Tn*5563*a isoform identified in *Pseudomonas* sp. LM7 in this study). The resolvases encoded by TIME-RES and Tn*5563*a are identical, and they contain a DNA binding helix-turn-helix (HTH) motif in their *C*-terminal parts that is highly conserved in this group of recombinases (data not shown). These observations indicate that TIME-RES is a hybrid element. It lacks a significant part of the core region of TIME, but has gained a site-specific recombination module. Therefore, TIME-RES may serve as a portable RES system.

TIME-COMP1 and TIME-COMP2 “captured” in the ZM1 strain, differ in structure from TIME-RES. These elements have been generated from two TIMEs and resemble typical IS-driven composite transposons. Thus, TIMEs are present at both ends of these TIME-COMP elements, placed either in the same (TIME-COMP2) or in the opposite orientation (TIME-COMP1) (Fig. 1A). The core regions of TIME-COMP elements are different, and they do not contain any genes involved in the process of transposition.

Transposition of TIME-COMP1 (2469 bp) and TIME-COMP2 (3892 bp) into the *sacB* gene resulted in the generation of 5-bp-long AT-rich DRs (Fig. 1C). As shown in Table 1, the transposition frequencies of these elements varied significantly. TIME-COMP2 showed very low transposition activity, since only a single pMAT1 derivative containing this element was detected among the tested pool of Suc^r^ clones.

The TIMEs flanking TIME-COMP1 are not identical. The 5′-end TIME is 100% identical to TIME1, while the 3′-end TIME (TIME4) differs in 1 nucleotide (cytosine to thymine transition at position 152) (Fig. 2A). In comparison, TIME-COMP2 contains identical copies of TIME4 at both ends.

The core region of TIME-COMP1 (1945 bp) contains two ORFs, coding for a putative resolvase TnpR (187 aa) (ORF1) and a putative protein of unknown function (167 aa), representing the DUF1652 family (ORF2). BLASTP analysis revealed that genes encoding DUF1652 proteins are conserved exclusively in the chromosomes of several *Pseudomonas* species, but they are not structurally linked with any TEs [highest similarity (99%) observed with a hypothetical protein of *Pseudomonas stutzeri* RCH2 (accession no. YP_007242478)]. The predicted TnpR of TIME-COMP1 shares the highest amino acid similarity (84%) with a putative resolvase of *P. stutzeri* (accession no. WP_019407077). Most of the related proteins are not transposon-encoded and they are shorter and more divergent than typical resolvases of the Tn*3*-family [e.g. TnpR of TIME-COMP1 (187 aa) displays only 45% aa identity to TnpR of TIME-RES (309 aa)].

*In silico* sequence analysis of the core region of TIME-COMP2 revealed the presence of four ORFs. Interestingly, the predicted protein encoded by ORF1 shows significant similarities to Rep proteins involved in the replication initiation of several plasmids: pPMA4326C of *Pseudomonas syringae* pv. maculicola ES4326 (74% aa identity) [36], pXF5823 of *Xylella fastidiosa* LAR20 (72%) [37] and pHLHK19 of *Laribacter hongkongensis* HLHK19 (52%) [38]. The other three ORFs of TIME-COMP2 encode putative proteins: (i) ORF2– hypothetical protein with 75% aa sequence similarity to a related protein of *P. aeruginosa* BWHPSA040 (accession no. ETV34345), (ii) ORF3– virulence-associated protein (VapD) found in many pathogenic bacterial strains with 76% aa identity to VapD of *Candidatus* Glomeribacter gigasporarum BEG34 (accession no. WP_006682113), and (iii) partial ORF4– MobA/L family protein involved in plasmid mobilization for conjugal transfer with 80% aa identity to *P. syringae* pv. glycinea str. race 4 (accession no. EGH16743).

Since the termini of TIMEs and TIME-derived elements share the highest level of sequence identity with the IRs of Tn*5563*a, it is probable that the Tn*5563*a-encoded transposase and similar transposases are responsible for mobilization of these non-autonomous elements. Analysis of the distribution of related transposase genes in the mine *Pseudomonas* spp. strains, performed by PCR (primers TNPR and TNPF; Table S2), identified homologous sequences in strains ZM1 and LM8 (in which transposition of TIMEs was observed) as well as in ZM2, LM5, LM6 and LM14. The amplified DNA fragments (1555 bp) encoded predicted proteins with 97–100% aa sequence identity to the transposase of Tn*5563*a, which proves wide distribution of related transposons (complete or truncated) in collected *Pseudomonas* spp. strains.

### TIMEs in sequence databases

Database searches by sequence similarity (BLASTN) were performed to identify elements related to TIMEs. Searches were made for highly similar as well as somewhat similar sequences. This analysis identified 18 complete TIMEs as well as several truncated forms containing either the 3′ or 5′ terminal parts of the elements. The vast majority of these sequences originate from *Pseudomonas* spp. strains, which strongly suggests a host preference of these elements. However, in two cases, TIMEs were identified in strains representing other genera of *Gammaproteobacteria*: *Proteus mirabilis* and *X. campestris* (Table S3).

TIMEs are found both in plasmids (11 elements) and bacterial chromosomes (7). They usually occur in close proximity to genes encoding transposases and rarely within defined Tn*3*-family transposons. Interestingly, only four of the identified elements are flanked by DR sequences (Table S3). Since a few of the identified TIMEs from both sites are flanked by 3′ terminal parts of different genes, it is highly probable that homologous recombination between two copies of these elements has occurred.

In contrast to other MITEs, TIMEs are not widely distributed within their hosts’ genomes. They are usually present as single copy elements. Two copies were found only in three cases: within plasmid QKH54 of an uncultured bacterium [39] and in the chromosomes of two strains of *Pseudomonas* spp. (accession nos. AB083212 and CP005976, respectively) (Table S3).

Comparative analysis of TIMEs revealed that all contain conserved terminal IR sequences, typical for the Tn*3*-family transposons, whose consensus sequence is presented in Fig. 2B. The core region of the elements is less well conserved. Four classes of TIMEs could be distinguished based on comparative analysis, differing in the level of sequence conservation (Fig. 2A). Interestingly, despite the observed sequence differences, the size of TIMEs is highly conserved. Majority are 262 bp long, with exception of two elements identified in chromosome of *P. putida* H8234 (261 bp) and an element identified in plasmid pDC3000A of *P. syringae* pv. tomato DC3000 (256 bp) (Table S3).

**Figure 3:**
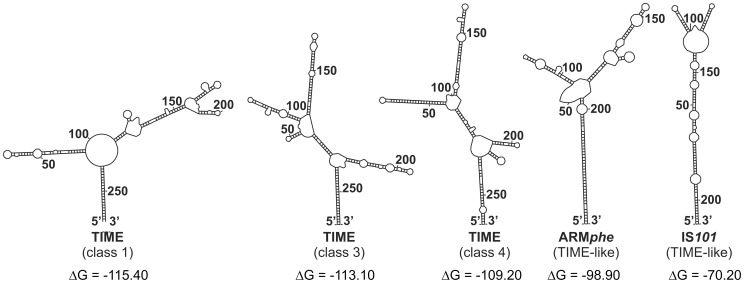
Predicted secondary structures of TIMEs at the RNA level. RNA secondary structures predicted by *in silico* folding using Mfold software for TIME1 (class 1), two TIMEs representing class 3 and 4 elements shown in Fig. 3 (accession nos. AF020724 and AE016855, respectively), and the TIME-like elements ARM*phe* and IS*101* (see Discussion for details). The minimum folding energy (ΔG) of the predicted secondary structures was calculated by Mfold.

As shown in Fig. 2A, all the identified TIMEs contain short stretches of conserved sequences in their core regions. Since these elements (as well as other MITEs) may be potentially co-transcribed together with adjacent genes, it is highly probable that such sequences may determine the tertiary structure of the resulting transcripts. Indeed, the results of Mfold analysis revealed that transcripts containing different TIMEs have the potential to fold into similar stable secondary structures [40] (Fig. 3), like those determined for other MITEs (e.g. [10]).

Analysis of the distribution of TIME derived transposon-like TEs in bacterial genomes revealed the presence of only one such element, identical to TIME-RES but without flanking DRs, within the chromosome of *P. stutzeri* DSM 4166 (accession no. CP002622; position 4333459–4334644).

### Distribution of TIME elements in collected bacterial strains

The distribution and copy number of TIMEs in the genomes of bacterial strains isolated from Lubin Mine and Zelazny Most was examined by DNA-DNA hybridization. DIG-labeled molecular probes specific for (i) TIMEs (PCR-amplified TIME fragment), (ii–iii) TIME-RES and TIME-COMP1 (*tnpR* gene fragments) and (iv) TIME-COMP2 (*rep* gene fragment) were hybridized with total DNA from 13 strains of *Pseudomonas* spp. that had been digested with restriction endonucleases.

This analysis revealed that TIME homologs are present in only four of the examined *Pseudomonas* spp. strains: ZM1 (3 TIME copies detected), LM8 (4 copies), LM14 (2 copies) and LM6 (1 copy) (data not shown). Since no TIMEs were “captured” in LM6 or LM14 using trap plasmid pMAT1, PCR analysis was used to determine whether these strains contain complete copies of the elements. A degenerate primer IRINEL, complementary to the IR sequences of TIMEs, was used in PCR with total DNAs of the aforementioned strains as template (DNA of ZM1 and LM8 served as control templates). In each case, a specific DNA product of the expected size was obtained. DNA sequencing confirmed the presence of intact TIME copies in strains LM6 and LM14 that were identical to TIME1 and TIME2, respectively.

The hybridization analysis also showed that the TIME-derived transposon-like elements are present at one copy per genome in the strains LM8 (TIME-RES) and ZM1 (TIME-COMP), in which they were previously identified using trap plasmid pMAT1.

Hybridization analysis was then used to determine the location (plasmid/chromosome) of TIME elements in the ZM1, LM8, LM6 and LM14 genomes. DNA electrophoretic analysis indicated that only two strains, ZM1 and LM8, contain plasmids, which have been designated pZM1P1 and pLM8P2, respectively. A positive hybridization signal with a TIME-specific molecular probe was observed with DNA fragments of both plasmids. The hybridization pattern suggested that they contain one (pLM8P2) or two (pZM1P1) TIME copies. Interestingly, both TIME-COMP1- and TIME-COMP2-specific probes hybridized with DNA of plasmid pZM1P1.

### Characterization of natural plasmids containing TIMEs

**Figure 4:**
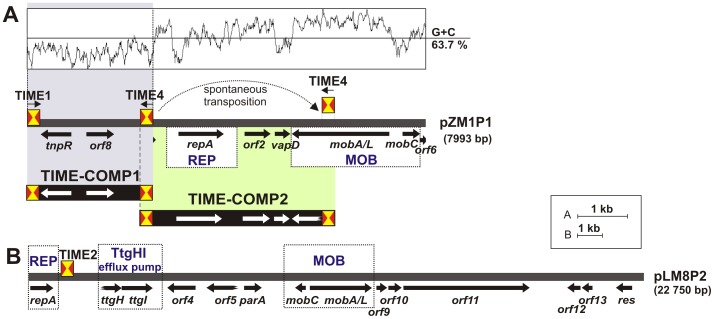
Genetic structure of *Pseudomonas* spp. plasmids containing non-autonomous TEs of the Tn*3* transposon family. Circular plasmids pZM1P1 (A) and pLM8P2 (B) originate from *Pseudomonas* spp. strains ZM1 and LM8, respectively. Predicted genetic modules involved in plasmid replication (REP), mobilization for conjugal transfer (MOB) and toluene resistance (TtgGHI efflux pump - truncated operon) are boxed and appropriately labeled. Predicted coding regions are represented by thick arrows indicating the direction of transcription. Broken arrows indicated truncated genes (*ttgH* and *orf5* of pLM8P2). Shaded areas connect DNA regions of pZM1P1 included in non-autonomous TIME-COMP transposons. Note that TIMEs are not drawn to scale. Thin black arrows above TIMEs indicate the orientation of the elements. A plot of the G+C content of pZM1P1 is shown above the structure diagram and the average G+C value is given to the right.

The nucleotide sequences of plasmids pZM1P1 (7793 bp) and pLM8P2 (22 750 bp) were determined and analyzed. The genetic organization of these plasmids is presented in Fig. 4. A summary of the identified ORFs, including their position, the size of the putative proteins they encode and their closest homologs, is presented in Table S4.

As shown in Fig. 4, a common feature of these plasmids is the presence of predicted genetic modules involved in (i) replication (REP) and (ii) mobilization for conjugal transfer (MOB). However, these replicons differ in their overall genetic organization. Plasmid pLM8P2 also contains a truncated *ttgGHI* solvent efflux pump operon. Homologous operons, commonly identified in *P. putida* strains, play a major role in the extrusion of toluene and a wide range of toxic aromatic hydrocarbons [41], [42].

The REP modules encode diverse replication initiation proteins, conserved in many *Pseudomonas* spp. plasmids. pZM1P1 contains ORF1 encoding a member of the Rep_3 superfamily (PFAM: PF01051), with highest similarity to a protein encoded by plasmid pPMA4326C from phytopathogenic strain *P. syringae* pv. maculicola ES4326 (74% aa sequence identity) (accession no. YP_025704) [36]. pLM8P2 encodes a protein most similar to RepA of *P. alcaligenes* plasmid pRA2 (65% aa sequence identity) (accession no. YP_025331) [43].

The results of the hybridization analysis indicated that pZM1P1 contains both TIME-COMP1 and TIME-COMP2. However, sequence analysis of this plasmid identified only a complete copy of TIME-COMP1 (Fig. 4A). This element is flanked by 5-bp long DRs (5′-AAGGA-3′), confirming that it was incorporated into the plasmid genome by means of transposition. It is located within a terminal part of ORF6 (disrupted upon insertion) encoding a predicted protein of unknown function. Analysis of the %GC profile of the pZM1P1 nucleotide sequence showed that the TIME-COMP1 DNA region has a significantly lower GC content (53.3%) than the rest of the plasmid (63.7%), suggesting its exogenous origin.

Interestingly, comparative sequence analysis revealed that the DNA region of pZM1P1 directly adjacent to TIME-COMP1 (Fig. 4A) is identical to the core region of TIME-COMP2. Therefore, the TIME-COMP2 copy, “captured” by a trap plasmid pMAT1, seems to have been generated by two transposition events: (i) spontaneous transposition of a TIME into another location within the pZM1P1 genome followed by (ii) TIME-directed mobilization for transposition of part of the pZM1P1 genome containing the functional REP module (Fig. 4A).

Plasmid pLM8P2 contains only one TIME, which is not bordered by DRs. In this case, the TIME is also placed close to the REP region, downstream of the gene encoding replication initiation protein RepA (Fig. 4B).

### Transcription activation by TIMEs

To test whether TIMEs contain a promoter capable of driving the transcription of downstream genes, a TIME1 element was amplified by PCR and inserted (in both orientations – termed A and B) into the broad host range promoter probe vector pCM132 (functional in *Pseudomonas* spp.) to generate transcriptional fusions with a promoter-less *lacZ* reporter gene (“A” orientation – IRR adjacent to the *lacZ* gene; “B” orientation – IRL adjacent to the *lacZ* gene). The constructed pCM132 derivatives (pCM132-TIME_A and pCM132-TIME_B) were introduced into *P. aeruginosa* PAO1161 and β-galactosidase activity assays were used to examine promoter strength.

Increased β-galactosidase activity was observed only in PAO1161 containing pCM132-TIME_A (141±14 Miller units, compared to 70±1 Miller units for the strain containing control plasmid pCM132). No increased activity was observed in the case of pCM132-TIME_B (data not shown). These results indicate the presence of a weak promoter within TIME1 (“A” orientation), but detailed inspection of the nucleotide sequence did not allow its localization.

Since the nucleotide sequences of the 3′-end parts of TIMEs and TIME-RES (104 bp; contains IRR) are highly conserved (Fig. 2), the terminal DNA region of TIME-RES was amplified by PCR, cloned into pCM132 and tested as described above. However, no promoter activity was detected in this case (data not shown).

## Discussion

**Figure 5:**
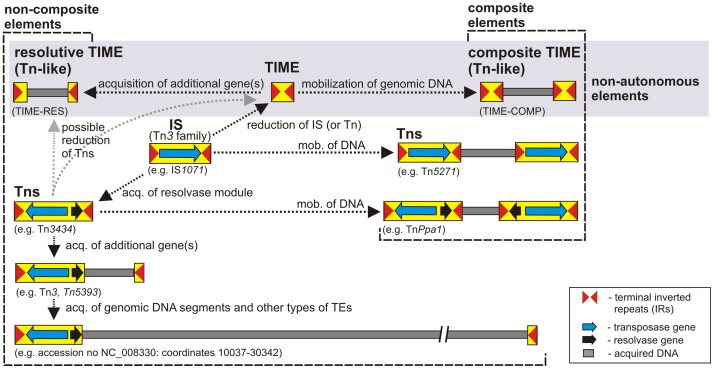
Possible mechanism for the generation of diverse non-autonomous and autonomous elements of the Tn*3* transposon family. The elements of the Tn3 family originate from progenitor insertion sequences (IS). Tns – diverse autonomous non-composite or composite transposons generated by acquisition of foreign DNA and mobilization for transposition of genomic DNA results. TIME - non-autonomous elements resulting from a reduction in the number of transposon-encoded genes, able to form mosaic elements resembling non-composite and composite Tns in structure. Elements representative of the different types are shown as examples: IS*1071* (accession no. M65135), Tn*3434* (accession no. AY232820), Tn*5393* (accession no. M96392), Tn*Ppa1* (accession no. DQ149577) and Tn*5271* (accession no. U18133).

In this study we identified and analyzed active transposable elements in a pool of strains of the genus *Pseudomonas*, isolated from Lubin copper mine and post-flotation tailings in Zelazny Most in Poland. A surprising discovery was the identification of several related TEs of diverse structure, which represented novel non-autonomous transposons (Fig. 5). Although these elements lack a transposase gene, they possess characteristics typical of the Tn*3* transposon family [44]. The simplest forms of the non-autonomous elements were designated TIMEs (Tn*3* family-derived Inverted-repeat Miniature Elements). All TIMEs identified in this study are of the same size (262 bp) and show a very high level of sequence similarity (99–100%). BLAST searches of the NCBI databases identified related elements with more divergent sequences (70–99% identity), but they all possessed identical IRs (Fig. 2).

Initiation of the process of transposition requires binding of a transposase to compatible terminal IRs. The ability of Tn*3*-family transposases to interact with IRs of other elements of this family has been reported previously [45]. The high level sequence identity of the IRs of TIMEs and Tn*5563*a, identified in this study (Fig. 1B), strongly suggests that Tn*5563*a-related transposases are responsible for the *trans*-mobilization of TIMEs. We did not observe transposition of Tn*5563*a in *Pseudomonas* spp. strains LM8 and ZM1 in which TIMEs were identified. However, the presence of genes encoding the predicted *trans*-acting transposases, with 99–100% aa identity to Tn*5563*a transposase, was demonstrated in both strains using PCR (data not shown). Both TIMEs and autonomous Tn*5563*a were found to transpose into trap plasmids at comparable frequencies, which proves that *trans*-mobilization of non-autonomous elements is not a rare phenomenon. We detected TIMEs in 4 of the analyzed strains of *Pseudomonas* spp., which strongly suggests that these elements might be more widely disseminated than expected from a bioinformatic database survey (18 elements identified in available bacterial genome sequences; Fig. 2; Table S3). Like autonomous transposons of the Tn*3* family, most TIMEs are present at one copy per genome. This may result from transposition immunity, a phenomenon that is thought to apply to members of the Tn*3* family, which precludes the transposition of more than one copy of an element into a single replicon [44].

The presence of conserved IRs within TIMEs permits the rapid identification of these elements irrespective of the sequence diversity of their core regions. Related elements, designated κγ and ΔxIS, have previously been identified by *in silico* analysis of nucleotide sequences originating from different strains of *Pseudomonas* spp., in the course of unrelated projects [46], [47]. The κγ element was identified in the Tn*3-*family transposon Tn*5041* of *Pseudomonas* sp. KHP41, within a 4-kb region containing relics of mobile elements and truncated genes [46], while the ΔxIS element was found close to the *pheAB* operon within plasmid pAM10.6 of *P. fluorescens* Cb36 [47]. As shown in Fig. 2 (AF020724), this element has the sequence characteristics of TIMEs.

Two other TIME-like elements have also been identified previously: ARM*phe* and IS*101*. Although they lack overall sequence similarities with TIMEs, their IR sequences are conserved in several Tn*3*-family transposons. The ARM*phe* element was identified together with ΔxIS by Peters and colleagues [47], who observed the presence of two short (240 bp), identical “IS-like elements” flanking the *pheAB* operon, which resembled the structure of a composite transposon.

The IS*101* element is of special interest because its functional characterization was performed during early studies on the Tn*3-*family transposons. It was designated as an insertion sequence, but it does not encode a transposase. This 209-bp-long element was discovered as a natural component of plasmid pSC101, by its ability to generate *in vivo* co-integrates between the parental replicon and filamentous phage f1, in a manner typical for replicative transposition [48]. Further studies revealed that IS*101* can be *trans-*mobilized for transposition by Tn*1000* transposase [49], and therefore it should be considered a MITE.

Interestingly, it was also demonstrated that IS*101* contains a resolution site, which can function – albeit poorly – with Tn*3*- and Tn*1000*-encoded resolvases [48]. Therefore, this element contains the *cis*-required regions that are crucial for both transposition (IRs) and efficient resolution of co-integrates (predicted *res* site with very limited sequence similarity to the Tn*3* or Tn*1000 res* sites [45]). We speculate that TIMEs as well as ARM*phe* elements may also contain functional *res* sites, but the identification of compatible resolvases is necessary to prove this.

All of the aforementioned elements originate from different progenitor Tn*3-*family transposons. Although their nucleotide sequences are divergent, they constitute a distinct group of elements. Therefore, we suggest the establishment of a TIME family, gathering all elements that can be mobilized for transposition by Tn*3*-family transposons. Both ARM*phe*, predicted *in silico*, and the functional IS*101* elements, which have arisen independently from TIMEs, should be included in this family. We suggest designating these elements TIME_ARM*phe*_ and TIME_IS*101*_, respectively.

TIME elements are found within both intergenic regions and annotated genes. Mfold analysis revealed that TIMEs are able to fold into long stem-loop structures at the RNA level, with free energies (ΔG) ranging from approx. −70 kcal/mol to −115 kcal/mol (Fig. 3), which is typical for many MITEs (e.g. [50]). Since their RNAs are predicted to form thermodynamically stable secondary structures, the co-transcription of TIMEs with nearby genes might affect the expression of these genes by changing their mRNA stability, e.g. as in the case of ERIC elements of *Enterobacteriaceae* [51]. This altered expression may result in various phenotypes, depending on the specific role of a given gene. In plasmid pLM8P2, whose structure was revealed in this study (Fig. 4B), the TIME is placed downstream of the *rep* gene encoding replication initiation protein. The possible co-transcript may have altered stability, which could potentially affect the rate of replication initiation and might consequently influence the copy number of the replicon.

Another interesting attribute of TIMEs demonstrated in this study, is their ability to mobilize segments of genomic DNA for transposition, which results in the generation of more complex non-autonomous elements (named TIME-COMP), resembling IS-driven composite transposons in structure. Moreover, we have shown that TIMEs may contain functional promoters, which could drive the transcription of downstream genes and potentially activate “silent phenotypes”. Therefore, TIMEs may play the role of a natural, transposition-based gene capture and expression system.

Using a trap plasmid, we demonstrated the transposition of such a transposon-like element (TIME-COMP1), originally residing in the natural plasmid pZM1P1 of *Pseudomonas* sp. ZM1. Interestingly, we also found that spontaneous transposition of a TIME into a separate location within this plasmid resulted in the generation of another element (TIME-COMP2), whose core region contains the pZM1P1 replication system.

It is well known that plasmid genomes can be separated into several DNA cassettes encoding specific functions. The observed huge diversity of plasmids is the result of recombinational shuffling of DNA segments (containing single genes or whole genetic modules) between different replicons co-residing in a cell. Here we provide the first evidence that such gene shuffling may be driven by non-autonomous TEs. Transposition events might therefore significantly influence the structure of bacterial plasmids, leading to the generation of molecules with novel properties, including composite plasmids containing more than one replication system (e.g. [52]).

Another type of functional non-autonomous element described in this study is represented by TIME-RES – a TIME derivative containing a genetic module homologous to the site-specific recombination system of the Tn*3*-family transposon Tn*5563* [32]. Recombination systems of this type play an important role in the biology and evolution of both transposable elements and plasmids. When present in transposons, they enable efficient resolution of co-integrates, i.e. intermediates of replicative transposition, while in plasmids they function as stabilization systems, responsible for the resolution of plasmid multimers into monomeric forms (MRS, multimer resolution system). Therefore TIME-RES (containing a predicted *tnpR* gene and *res* site) most probably serves as a portable recombination system, whose insertion may significantly modify the properties of different mobile genetic elements.

TEs of the Tn*3* family are ubiquitous in bacterial genomes. It is believed that the Tn*3*-family transposons originated from progenitor IS elements that acquired an additional genetic module responsible for site-specific recombination [53] (e.g. by insertion of an TIME-RES-like element). This evolutionarily-advantageous event initiated the acquisition of further genes whose products could directly affect the phenotype of their host.

For many years the Tn*3* family of elements was thought to be comprised of (i) insertion sequences (30 known elements; ISfinder database), (ii) composite transposons generated by ISs (e.g. Tn*5271* driven by two copies of IS*1071* [53]), and (iii) a large group of non-composite transposons of two evolutionary lineages (Tn*3-* and Tn*501-*subfamilies). Our studies performed with trap plasmids have demonstrated that this family is much more diverse than previously thought. It also includes a composite transposon generated by two copies of a non-composite cryptic transposon Tn*3434* [1], [54] and, as described in this study, simple non-autonomous elements (TIMEs) and their derivatives with structures typical for composite transposons (TIME-COMP) and non-composite transposons (TIME-RES) (Fig. 5). Moreover, bioinformatic analyses of the nucleotide sequences of several bacterial genomes revealed the presence of large elements, originating from Tn*3*-family non-composite transposons, that have acquired additional genetic information, including numerous metabolic genes and other types of transposable element, thus forming transposable genomic islands (Fig. 5). Therefore, the Tn*3-*family includes representatives of all types of bacterial transposable element that have been described so far. Similar diversity is also observed in case of IS derived elements [55].

Bioinformatic sequence analysis also permitted the identification of many solo Tn*3*-family IR-like sequences. Taking into account the ability of some Tn*3*-family transposons to transpose *via* one-ended transposition (producing different random endpoints at one end of the transposed element) [56], it is probable that even single IR sequences can enable the generation of as yet unidentified non-autonomous TE types.

## Supporting Information

Table S1 *Pseudomonas* spp. strains isolated from black shale ore of Lubin mine (LM) and postflotation tailings in Zelazny Most (ZM) used in this study.
(DOC)

Table S2 Oligonucleotide primers used in this study.
(DOC)

Table S3 Identification of TIME-like elements in GenBank database.
(DOC)

Table S4 ORFs located within the plasmids pZM1P1 and pLM8P2 analyzed in this study.
(DOC)
