# Supplementary material for: Mobility and Generation of Mosaic Non-Autonomous Transposons by Tn3-Derived Inverted-Repeat Miniature Elements (TIMEs)
Source: PLoS One. 2014 Aug 14;9(8):e105010. doi: 10.1371/journal.pone.0105010 (PMC4133298; doi:10.1371/journal.pone.0105010)
Supplement: Table S1 — Pseudomonas spp. strains isolated from black shale ore of Lubin mine (LM) and postflotation tailings in Zelazny Most (ZM) used in this study. (DOC) [file pone.0105010.s001.doc]

**Table S1.** *Pseudomonas* spp. strains isolated from black shale ore of Lubin mine (LM) and postflotation tailings in Zelazny Most (ZM) used in this study.

| ***Pseudomonas* spp. strains** | **GenBank accession number** |
| --- | --- |
| *Pseudomonas* sp. ZM1 | KJ765933 |
| *Pseudomonas* sp. ZM2 | KJ765934 |
| *Pseudomonas* sp. LM5 | EU821341 |
| *Pseudomonas* sp. LM6 | EU821342 |
| *Pseudomonas mendocina* strain LM7 | EU821343 |
| *Pseudomonas* sp. LM8 | EU821344 |
| *Pseudomonas* *aeruginosa* strain LM10 | KF769955 |
| *Pseudomonas* sp. LM11 | KF769956 |
| *Pseudomonas* sp. LM12 | KF769957 |
| *Pseudomonas* sp. LM13 | KJ765935 |
| *Pseudomonas* sp. LM14 | KF769958 |
| *Pseudomonas* sp. LM15 | KF769959 |
| *Pseudomonas* sp. LM25 | KF769969 |
