# Supplementary material for: Mobility and Generation of Mosaic Non-Autonomous Transposons by Tn3-Derived Inverted-Repeat Miniature Elements (TIMEs)
Source: PLoS One. 2014 Aug 14;9(8):e105010. doi: 10.1371/journal.pone.0105010 (PMC4133298; doi:10.1371/journal.pone.0105010)
Supplement: Table S2 — Oligonucleotide primers used in this study. (DOC) [file pone.0105010.s002.doc]

**Table S2. Oligonucleotide primers used in this study.**

| **TE** | **Primer** | **Sequence (5’→3’)** |
| --- | --- | --- |
| **Primers used for the PCR amplification of hybridization probes** | | |
| TIME | IRINEL | 5’-GGGGTTTGGGGAGCARTGGAACCAA-3’ |
| INEL1 | 8M21Fs | 5’-ATCTCGGTCAGCAGGTCATG-3’ |
|  | 8M21Rs | 5’-GCGAACGCATCTACCAAGAC-3’ |
| INEL2 | SINELFcd | 5’-AACCGATTGGCCTTCCTCGA-3’ |
|  | resINELRs | 5’-GATCTGGAGCAACAACGCCT-3’ |
| **Primers used for the identification of promoters within TIME1 and INEL1** | | |
| TIME1 | PIMEL | 5'-gcgaattcCGTACCTGATGCAGGAAGCC-3' |
|  | PIMER | 5'-gtggatccCGTTCATCGAGCCACGCTAT-3' |
|  | PIME-L | 5'-gtggatccCGTACCTGATGCAGGAAGCC-3' |
|  | PIME-R | 5'-gcgaattcCGTTCATCGAGCCACGCTAT-3' |
| INEL1 | INELL | 5'-gcgaattcGAGCCATTCCTCGATAGAGT-3' |
|  | INELR | 5'-gtggatccCCTCTACACCGTTGATGTTG-3' |
|  | INEL-L | 5'-gtggatccCCTCTACACCGTTGATGTTG-3' |
|  | INEL-R | 5'-gcgaattcGAGCCATTCCTCGATAGAGT-3' |
