# Supplementary material for: Mobility and Generation of Mosaic Non-Autonomous Transposons by Tn3-Derived Inverted-Repeat Miniature Elements (TIMEs)
Source: PLoS One. 2014 Aug 14;9(8):e105010. doi: 10.1371/journal.pone.0105010 (PMC4133298; doi:10.1371/journal.pone.0105010)
Supplement: Table S3 — Identification of TIME-like elements in GenBank database. (DOC) [file pone.0105010.s003.doc]

**Table S3. Identification of TIME-like elements in GenBank database.**

| **Host strain** | **GenBank accesion no.** | **Location:**  **plasmid/**  **chromosome (nucleotide position)** | **Insertion site** | **Length (bp)/**  **number of conserved residues (identical with TIME1)** | **DR**  **(bp; sequence**  **5’→3’)** | **Copy number in the genome** |
| --- | --- | --- | --- | --- | --- | --- |
| *Pseudomonas putida* DOT-T1E | **HM626202** | plasmid pGRT1  (108410-108671) | 3’-terminal part of gene 1 (hypothetical protein)  3’-terminal part of gene 2  (putative transcription regulator protein) | 262/261 | N | 1 |
| *Pseudomonas putida* W619 | **CP000949** | chromosome  (2609011-2608750) | 3’-terminal part of gene 1  (transcriptional regulator, LysR family)  3’-terminal part of gene 2  (integrase) | 262/261 | N | 1 |
| *Proteus mirabilis* | **DQ352175** | plasmid R772  (2046-1785) | 3’-terminal part of gene 1  (putative transcription regulator protein)  3’-terminal part of gene 2  (hypothetical protein) | 262/261 | N | 1 |
| Uncultured bacterium from freshwater  environments | **AM157767** | plasmid QKH54  (12549-12288) | IGR | 262/261 | 5; ATCAC | 2 |
|  |  | plasmid QKH54  41496-41757 | IGR | 262/261 | 4; TACT |  |
| *Xanthomonas campestris* pv. *vesicatoria* | **AM039952** | chromosome  (2679718-2679979) | 5’-terminal part of gene 1  (putative transposase)  3’-terminal part of gene 2  (putative major facilitator superfamily protein) | 262/261 | N | 1 |
| *Pseudomonas putida* | **AJ344068** | plasmid pWW0  (25441-25702) | 3’-terminal part of gene 1  (hypothetical protein)  3’-terminal part of gene 2  (putative transcription regulator protein) | 262/261 | N | 1 |
| *Pseudomonas stutzeri* | **PSU80214** | plasmid pBP  (5206-5467) | 3’-terminal part of gene | 262/259 | N | 1 |
| *Pseudomonas* sp. KHP41 | **X98999** | transposon Tn*5041*  (5417-5678) | IGR | 262/179 | N | 1 |
| *Pseudomonas aeruginosa*  AR-2 | **AB083212_A** | chromosome  (1120-859) | IGR | 262/143 | 2; TA | 1 |
|  | **AB083212_B** | chromosome  (7557-7297) | IGR | 262/142 | N | 1 |
| *Pseudomonas aeruginosa* | **AB120321** | chromosome  (8753-8492) | IGR | 262/143 | N | 1 |
| *Pseudomonas putida* H8234 | **CP005976_A** | chromosome  (3473571-3473831) | 3’-terminal part of gene (hypothetical protein) | 261/173 | N | 1 |
|  | **CP005976_B** | chromosome  (3502909-3502649) | 5’-terminal part of gene 1 (integrase)  5’-terminal part of gene 2  (cytochrome C) | 261/144 | 5; AAATA | 1 |
| *Pseudomonas putida* | **AF130439** | plasmid pPGH2  (4099-3838) | IGR | 262/154 | N | 1 |
| *Pseudomonas putida* | **FJ859895** | plasmid pAK5  (150-411) | IGR | 262/180 | N | 1 |
| *Pseudomonas putida* GJ31 | **AY831462** | plasmid pKW1  (4814-5075) | IGR | 262/180 | N | 1 |
| *Pseudomonas fluorescens*  Cb36 | **AF020724** | plasmid pAM10.6  (ND) | ND | 262/180 | N | 1 |
| *Pseudomonas syringae* pv. *tomato* DC3000 | **AE016855** | plasmid pDC3000A  (31379-31634) | IGR | 256/180 | N | 1 |

ND – not determined

IGR – intergenic region

N – not present
