# Supplementary material for: Mobility and Generation of Mosaic Non-Autonomous Transposons by Tn3-Derived Inverted-Repeat Miniature Elements (TIMEs)
Source: PLoS One. 2014 Aug 14;9(8):e105010. doi: 10.1371/journal.pone.0105010 (PMC4133298; doi:10.1371/journal.pone.0105010)
Supplement: Table S4 — ORFs located within the plasmids pZM1P1 and pLM8P2 analyzed in this study. (DOC) [file pone.0105010.s004.doc]

**Table S4. ORFs located within the plasmids pZM1P1 and pLM8P2 analyzed in this study.**

| **ORFs located within plasmid pZM1P1 of *Pseudomonas* sp. ZM1** | | | | | | | |
| --- | --- | --- | --- | --- | --- | --- | --- |
| ORF no. | Coding region (bp) | Orientation | Protein size (aa) | Possible function | Best BLAST hits | | |
|  |  |  |  |  | Percentage identity (aa) | Organism | GenBank accession no. |
| 1 | 1-846 | → | 281 | replication protein (**RepA**) | 74 (207/278) | *Pseudomonas syringae* pv. maculicola ES4326 (plasmid pPMA4326C) | YP_025704 |
| 2 | 1291-1786 | → | 165 | hypothetical protein | 92 (113/123) | *Pseudomonas aeruginosa* 3579 | EZN88946 |
| 3 | 1848-2135 | → | 95 | putative virulence-associated protein D (**VapD**) | 99 (92/93) | *Pseudomonas aeruginosa* 3579 | EZN88945 |
| 4 | 2138-4159 | ← | 673 | mobilization protein A/L (**MobA/MobL**) | 93 (628/673) | *Pseudomonas aeruginosa* 3579 | EZN88944 |
| 5 | 4339-4620 | → | 93 | mobilization protein C (**MobC**) | 82 (75/92) | *Pseudomonas aeruginosa* 3579 | EZN88943 |
| 6 | 4620-4826 | → | 68 | hypothetical protein | 53 (27/51) | *Erwinia tasmaniensis* Et1/99 | YP_001909423 |
| 7 | 5057-5620 | ← | 187 | resolvase (**TnpR**) | 98 (184/187) | *Pseudomonas stutzeri* | WP_019407077 |
| 8 | 6009-6512 | → | 165 | hypothetical protein | 99 (95/96) | *Pseudomonas stutzeri* RCH2 | YP_007242478 |
| **ORFs located within plasmid pLM8P2 of *Pseudomonas* sp. LM8** | | | | | | | |
| ORF no. | Coding region (bp) | Orientation | Protein size (aa) | Possible function | Best BLAST hits | | |
|  |  |  |  |  | Percentage identity (aa) | Organism | GenBank accession no. |
| 1 | 1-810 | → | 269 | replication protein (**RepA**) | 65 (180/276) | *Pseudomonas alcaligenes* NCIB 9867  (plasmid pRA2) | YP_025331 |
| 2 | 2006-2617* | → | 203 | toluene efflux pump outer membrane protein (**TtgH**) | 96 (200/203) | *Pseudomonas* sp. Chol1 | WP_008568006 |
| 3 | 2614-4038 | → | 474 | toluene efflux pump outer membrane protein (**TtgI**) | 92 (440/464) | *Pseudomonas* sp. Chol1 | WP_008568005 |
| 4 | 5703-4642 | ← | 353 | acetyltransferase | 67 (234/348) | *Pseudomonas stutzeri* | WP_021209626 |
| 5 | 7242-6055* | ← | 395 | sulfate transporter | 89 (328/369) | *Pseudomonas stutzeri* CCUG 29243 | YP_006459742 |
| 6 | 7447-8079 | → | 210 | partitioning protein A (**ParA**) | 92 (193/210) | *Burkholderia pseudomallei* PHB194  (plasmid pPHB194) | YP_006960785 |
| 7 | 9629-9285 | ← | 104 | mobilization protein C  (**MobC**) | 48 (36/75) | *Pseudomonas* sp. GLE121  (plasmid pGLE121P1) | YP_007974357 |
| 8 | 9807-12011 | → | 93 | mobilization protein A/L (**MobA/MobL**) | 48 (95/197) | *Enterobacter cloacae* CHE-2  (plasmid pCHE-A) | YP_002563156 |
| 9 | 12127-12438 | → | 103 | transcriptional regulator | 68 (67/99) | *Gloeobacter kilaueensis* JS1 | YP_008710953 |
| 10 | 12441-12941 | → | 166 | hypothetical protein | 66 (121/160) | *Pseudomonas pseudoalcaligenes* CECT 5344 | CDM42352 |
| 11 | 13002-17894 | → | 1630 | helicase | 90(1469/1631) | *Pseudomonas stutzeri* CCUG 29243 (plasmid pAOVO02) | YP_006459665 |
| 12 | 19914-19462 | ← | 150 | hypothetical protein | 53 (79/148) | *Pseudomonas aeruginosa* | WP_023090290 |
| 13 | 20339-19923 | ← | 138 | hypothetical protein | 66 (92/139) | *Pseudomonas stutzeri* RCH2 | YP_007242140 |
| 14 | 22020-21427 | ← | 197 | resolvase | 86 (163/189) | *Sinorhizobium meliloti* SM11  (plasmid pSmeSM11b) | YP_001965616 |

* - incomplete gene
